# Climate Change Reshapes Thermal Suitability for Dairy Cattle in Jiangsu Province (1961–2020)

**DOI:** 10.3390/ani16081166

**Published:** 2026-04-10

**Authors:** Guangyi Yang, Fei Liu, Guangqin Zhu, Qiong Liu, Chao Wang, Dong Li, Zhongrui Guo, Hongmei Zhao

**Affiliations:** 1 School of Ecology and Environment, Xuzhou Vocational College of Bioengineering, Xuzhou 221006, China; guangyiyang1993@163.com (G.Y.); 18105229500@163.com (F.L.);; 2 Northeast Institute of Geography and Agroecology, Chinese Academy of Sciences, Changchun 130102, China; 3 University of Chinese Academy of Sciences, Beijing 100049, China

**Keywords:** thermal stress, temperature–humidity index, spatiotemporal analysis, forecasting platform, dairy cattle

## Abstract

Understanding how climate change alters the thermal environment experienced by dairy cattle is critical for guiding adaptation in rapidly warming regions. Using meteorological data from 1961 to 2020, this study quantified long-term trends in temperature, precipitation, relative humidity, and wind speed across Jiangsu Province, China, and assessed their impacts on thermal stress using a temperature–humidity index (THI). The results reveal pronounced spatial heterogeneity in climatic change, with accelerated warming in southern and coastal prefectures, and continued winter cold stress in the northern plain. Over the past six decades, the annual number of heat-stress days (THI > 72) increased substantially and expanded northward, while cold-stress days (THI ≤ 38) declined but remained non-negligible in northern Jiangsu. Although the total number of thermally comfortable days changed little at the provincial scale, their seasonal distribution became increasingly compressed between longer summer heat-stress periods and shorter winter cold-stress windows, indicating a narrowing of the effective comfort range for dairy cattle. To link historical analysis with operational applications, a forecasting platform was developed to generate short-term predictions of THI and associated meteorological conditions, enabling spatially explicit visualization and early identification of periods with elevated thermal risk. Overall, these findings demonstrate an intensification and redistribution of thermal stress in Jiangsu, while also illustrating a transferable climate-risk mechanism relevant to other warming, humid dairy regions worldwide.

## Simple Summary

Climate change is altering the environmental conditions experienced by dairy cattle and is creating new challenges for animal health and farm management. This study examined how the climate of Jiangsu Province, China, changed from 1961 to 2020 and how these changes affected the thermal suitability of dairy cattle. We found that heat-stress days increased markedly and spread northward, while cold-stress days decreased but still remained important in northern areas. Although the total number of comfortable days changed little, the period of suitable conditions became shorter because of longer hot periods and shorter cold periods. To help farmers respond to these risks, we also developed a short-term forecasting platform that can identify periods of thermal stress. These results can support better farm management and improve climate adaptation in dairy production.

## 1. Introduction

Livestock production is closely linked to climatic conditions because animal health, productivity, and welfare depend on the balance between metabolic heat production and environmental heat exchange [1,2]. Among livestock species, dairy cattle are particularly sensitive to thermal conditions due to their high metabolic heat load and relatively narrow thermoneutral range [3,4,5]. Variations in air temperature, relative humidity, wind speed, and precipitation jointly influence heat dissipation, feed intake, and physiological stability. In recent decades, climate change has altered not only mean temperature levels but also the frequency, duration, and seasonal timing of thermal extremes, increasing the likelihood that dairy cattle are exposed to thermal stress [6,7]. Understanding how these changes affect the temporal and spatial distribution of thermal stress is therefore a prerequisite for climate-informed dairy management.

Heat stress occurs when environmental heat load exceeds the animal’s capacity for sensible and evaporative heat loss, leading to elevated respiration rates, reduced feed intake, and declines in milk yield. Prolonged heat stress has been shown to impair reproductive performance, compromise immune function, and increase mortality risk [8,9,10]. Numerous studies have documented the negative impacts of heat stress on dairy production across both tropical and temperate regions, and climate projections consistently indicate that heat-stress exposure is likely to intensify in many dairy-producing areas [2,11]. In contrast, cold stress has received comparatively less attention in recent livestock–climate research, despite remaining an important constraint in regions influenced by strong winter monsoons or continental cold air outbreaks. When ambient conditions fall below the lower critical temperature, dairy cows must increase metabolic heat production to maintain thermal balance, resulting in higher energy requirements and reduced production efficiency [12]. Cold stress may be further exacerbated by high wind speeds and humidity, which increase convective and evaporative heat loss. In regions where hot summers and cold winters coexist, alternating exposure to heat and cold stress can impose cumulative physiological demands on dairy cattle. However, compared with heat stress, the long-term spatiotemporal dynamics of cold stress and its interaction with ongoing climate change remain insufficiently quantified.

Jiangsu Province, located in eastern China, is characterized by a humid subtropical monsoon climate with hot, humid summers and cold, often windy winters. Climatic conditions vary markedly across the province, with warmer and more humid environments in the south and along the coast, and colder winter conditions with stronger winds in the northern plain. Observational records and previous studies indicate that the regional climate has undergone notable changes over recent decades, accompanied by shifts in humidity, wind conditions, and the occurrence of extreme temperature events [13,14,15]. Jiangsu is also a major dairy-producing and milk-consuming region, where dairy systems have increasingly shifted toward intensive, facility-based production. Under such systems, animal performance is closely coupled with ambient environmental conditions, and both summer heat stress and winter cold stress can directly influence housing requirements, energy use, and seasonal management practices.

Previous studies on livestock–climate interactions in China have primarily examined temperature trends or heat-stress conditions derived from temperature–humidity indices [16,17]. In comparison, less attention has been given to the long-term spatial redistribution of both heat and cold stress and to their seasonal coexistence across regions. In addition, although global- and national-scale studies have projected future changes in thermal stress using climate models [18,19], relatively few investigations have reconstructed the historical evolution and spatial redistribution of thermal stress exposure at regional scales based on long-term meteorological records. Historical analyses are also rarely integrated with short-term assessment or forecasting tools that could support operational decision-making.

To address these gaps, the objective of this study is to provide a comprehensive assessment of the long-term spatiotemporal changes in thermal stress in dairy cattle across Jiangsu Province and to develop a forecasting platform that links historical climate analysis with short-term prediction. Specifically, this study reconstructs the long-term redistribution of both heat and cold stress over the period 1961–2020 and identifies the emergence of a dual-stress pattern, in which thermal stress affects different regions at different times under climate change. In addition, a forecasting platform is developed to enable short-term, spatially explicit prediction of thermal stress, thereby connecting historical climatic patterns with practical decision-making in dairy management. By combining long-term climate analysis with operational forecasting, this study provides an integrated framework for understanding thermal stress dynamics in dairy cattle and offers practical support for climate-adaptive management. The methodological approach is also transferable to other regions with similar climatic conditions, contributing to broader efforts to adapt dairy production systems to climate variability.

## 2. Methodology

### 2.1. Study Area

**Figure 1:**
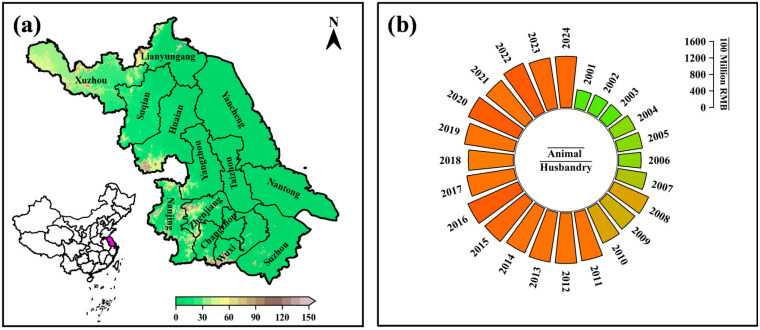
(**a**) Geographic location of Jiangsu Province and its administrative divisions; (**b**) output value of animal husbandry in Jiangsu Province during 2001–2024 (RMB 100 million).

Jiangsu Province is located in eastern China (30°45′–35°20′ N, 116°18′–121°57′ E), bordered by the Yellow Sea to the east and the Yangtze River to the south (Figure 1a). The province covers approximately 107,200 km^2^ and is characterized by a gentle topographic gradient sloping from northwest to southeast. Jiangsu has a humid subtropical monsoon climate with four distinct seasons. The mean annual temperature is approximately 15 °C, and mean annual precipitation is around 1000 mm, with the majority occurring between June and September. Climatic conditions vary across the province: southern cities such as Nanjing, Suzhou, Wuxi, and Changzhou are generally warmer and more humid, whereas northern areas including Xuzhou, Suqian, and Lianyungang experience colder winters and higher average wind speeds. Observational records indicate that the province has experienced notable warming since the 1960s, accompanied by changes in the frequency of extreme temperature events, while winter cold surges continue to occur, particularly in northern regions.

Animal husbandry is an important component of Jiangsu’s agricultural sector. As illustrated in Figure 1b, the output value of animal husbandry has increased steadily since 2000, exceeding 150 billion RMB by 2023 [20]. Dairy cattle, along with pigs and poultry, represent the main livestock species in the province. Dairy production is primarily concentrated in central and southern Jiangsu, where feed availability, infrastructure, and market access are relatively favorable. At the same time, the predominance of intensive production systems means that livestock performance is closely linked to ambient environmental conditions. Seasonal variations in temperature, humidity, and wind therefore provide a relevant background for examining spatial and temporal patterns of thermal stress affecting dairy cattle across the province.

### 2.2. Meteorological Data

This study utilizes the China Meteorological Forcing Dataset version 2.0 (CMFD v2.0, available online: https://data.tpdc.ac.cn/home, accessed on 31 July 2025), a long-term, high-resolution gridded dataset specifically designed to support land–atmosphere interaction research across China [21]. The dataset provides multiple near-surface meteorological variables essential for environmental modeling, including air temperature, precipitation, relative humidity, and wind speed—key inputs for evaluating climate suitability for livestock. The CMFD dataset spans the period from 1951 to 2024, with a spatial resolution of 0.1° (~10 km) and a temporal resolution of 1 day, enabling fine-scale temporal and spatial assessments across large regions. CMFD v2.0 is generated through the integration of ERA5 reanalysis products from the European Centre for Medium-Range Weather Forecasts (ECMWF), Reading, UK, with in situ observations from the China Meteorological Administration, Beijing, China.

In this study, we extracted and processed four key meteorological variables—daily mean temperature, precipitation, relative humidity, and wind speed—from the CMFD product to characterize the climatic environment relevant to dairy cattle and to calculate heat-stress, cold-stress, and no-stress days. These variables were extracted on a grid-by-grid basis, and trends were analyzed using linear slope fitting and Mann–Kendall (MK) tests to assess temporal variations. For each grid cell, the number of heat-stress days, cold-stress days, and no-stress days was calculated using established THI thresholds (Table 1). City-level data were derived by extracting grid cells corresponding to each city’s boundaries using the Python (version 3.11) Geopandas library, and averages were computed to determine the total number of heat-stress, cold-stress, and no-stress days.

### 2.3. Calculation of Heat and Cold Stress for Dairy Cattle

Thermal stress in dairy cattle was evaluated using the temperature–humidity index (THI), which integrates the effects of ambient temperature and relative humidity to quantify the degree of thermal load. The index is one of the most widely applied bioclimatic indicators in dairy cattle research, enabling direct comparability with existing studies across China and globally [22,23,24]. Although indices incorporating additional environmental variables such as solar radiation and wind speed can provide a more physically detailed representation of heat load, and more recent formulations have been proposed to better characterize heat exchange processes [25], such approaches are typically applied in short-term or site-specific physiological assessments. In contrast, the objective of this study is to investigate long-term spatiotemporal patterns of thermal stress across a large regional domain. In this context, the use of a standardized and widely validated index is more appropriate for ensuring consistency and comparability across both time and space. Wind speed in this study was analyzed separately to evaluate its role in modulating thermal stress, but was not integrated into the THI calculation itself. The combined effects of temperature, humidity, and wind speed were therefore considered in the broader interpretation of the thermal environment. Despite its known limitations, the THI remains a robust and practical indicator for large-scale and long-term climatological assessments of thermal stress. The THI was calculated according to the following equation [26]:

THI=(1.8×T)+32−[(0.55−0.0055×RH)×((1.8×T)−26.8)]

where T is the air temperature (°C) and RH is the relative humidity (%). This formulation was applied to daily gridded meteorological data across Jiangsu Province to obtain a continuous THI time series.

Based on established THI thresholds for dairy cattle and considering climatic conditions in eastern China, thermal conditions were classified into heat-stress, cold-stress, and no-stress states (Table 1). In this study, the heat-stress threshold of THI > 72 follows the widely used definition in dairy cattle studies, indicating the point at which evaporative cooling becomes progressively constrained [2]. The cold-stress threshold of THI ≤ 38 is adopted from Xu [27], below which dairy cattle increase metabolic heat production to maintain thermal balance. Days with 38 < THI ≤ 72 were considered no-stress days, representing thermally comfortable conditions for dairy cattle. For each grid cell and year, the annual number of heat-stress days and cold-stress days was calculated to quantify the duration of thermal stress exposure. These indicators were used to analyze long-term temporal trends and spatial patterns of thermal stress across Jiangsu Province.

### 2.4. Platform Framework for Short-Term Thermal Stress Forecasting

**Figure 2:**
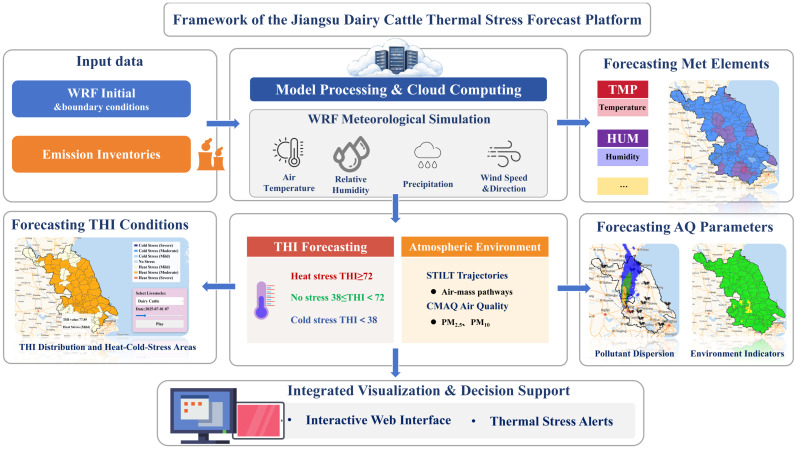
Framework of the Jiangsu Dairy Thermal Stress Forecast Platform based on WRF-STILT-CMAQ integration.

To enable short-term assessment of thermal stress conditions for dairy cattle, a Jiangsu Dairy Thermal Stress Forecast Platform was developed by integrating the Weather Research and Forecasting (WRF) model, the Stochastic Time-Inverted Lagrangian Transport (STILT) model, and the Community Multiscale Air Quality (CMAQ) model. The platform combines numerical weather prediction, THI-based thermal stress computation, and atmospheric transport modeling within a unified technical framework (Figure 2). 

The WRF model provides the meteorological forcing for the platform. It was configured to simulate near-surface air temperature, relative humidity, wind speed, and precipitation over Jiangsu Province at a horizontal resolution of 9 km. Model outputs were generated on a rolling basis to produce short-term forecasts up to 48 h. The forecast temperature and relative humidity fields were subsequently used to calculate THI values at each grid cell. Based on the forecast THI fields, thermal conditions were classified into heat-stress (THI > 72), cold-stress (THI ≤ 38), and no-stress conditions (38 < THI ≤ 72). Spatial distributions of thermal stress were generated at the grid scale and further aggregated to administrative (county-level) units for visualization.

Atmospheric transport processes were characterized using a STILT-based trajectory module driven by the WRF meteorological fields. Forward air-mass trajectories were simulated from selected locations to represent short-term airflow pathways and dispersion patterns under forecast meteorological conditions. In addition, the CMAQ model was coupled offline with WRF outputs to generate short-term simulations of ambient air-quality indicators, including PM_2.5_ and NO_2_. These air-quality fields were produced using the same meteorological forcing as the thermal stress calculations and were visualized alongside the THI-based stress maps. Air-quality variables were not included in the THI calculation and were treated as supplementary environmental information.

## 3. Results

### 3.1. Spatiotemporal Trends of Climatic Variables in Jiangsu Province (1961–2020)

**Figure 3:**
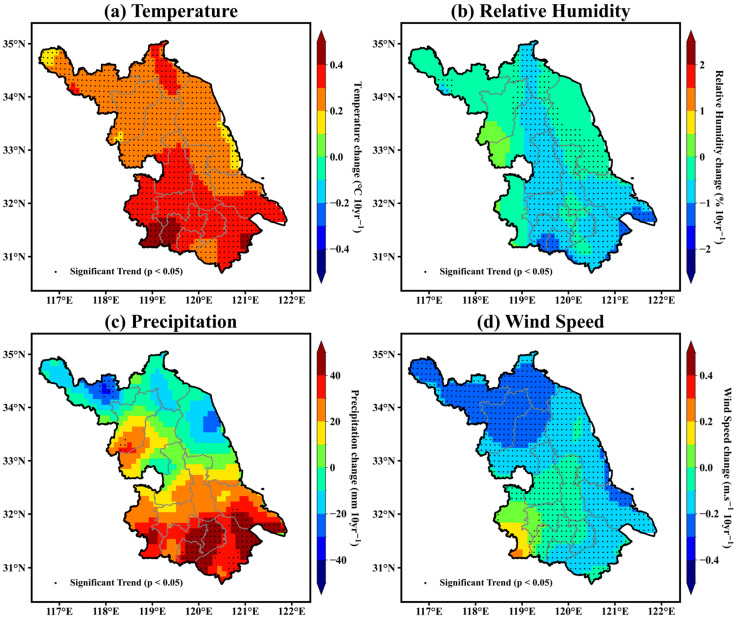
Decadal trends of key climatic variables in Jiangsu Province during the period 1961–2020: (**a**) mean temperature (°C), (**b**) relative humidity (%), (**c**) precipitation (mm), and (**d**) wind speed (m/s).

To investigate the long-term spatial patterns of climate change across Jiangsu Province, Figure 3 presents the decadal linear trends of four key meteorological variables (mean temperature, relative humidity, precipitation, and wind speed) over the period 1961 to 2020, based on pixel-wise linear regression. In addition, the statistical significance of these trends was evaluated using the Mann–Kendall (MK) test, with significant trends (*p* < 0.05) indicated in the figure. These climatic factors are crucial determinants of livestock growth, health, and productivity. The results reveal obvious spatial heterogeneity in both the direction and magnitude of change, highlighting the complexity of regional climate dynamics that underpin the suitability of livestock farming zones.

Warming dominates the climatic signal across Jiangsu, with the most rapid increases occurring in the southern and coastal subregions (Figure 3a). Most warming trends are statistically significant, particularly in the southern and central regions, indicating a robust and spatially coherent increase in temperature. The rise in mean temperature—approximately 0.3–0.5 °C per decade in the south and 0.2–0.3 °C per decade in the north—reflects both the influence of large-scale global warming and the enhancing effects of urban heat islands and land-use change. This north–south contrast suggests an interplay between anthropogenic and natural drivers, where intensified urbanization, reduced vegetation cover, and increased surface heat storage collectively intensify local warming [28,29]. Such warming has direct physiological implications for dairy cattle, as the nonlinear response of the THI amplifies even small temperature increases, pushing the thermal environment toward or beyond stress thresholds for longer durations each year. Although mean relative humidity (Figure 3b) has generally decreased, the spatial pattern is distinctly heterogeneous. The Mann–Kendall (MK) test indicates that statistically significant declines are concentrated along a central north–south band and in the eastern and southeastern coastal regions, whereas trends in parts of western Jiangsu are weaker, are non-significant, or locally exhibit slight increases. This tendency does not alleviate the increasing thermal load. The southern part of Jiangsu remains persistently humid during the monsoon months due to moisture advection from the East China Sea, maintaining near-saturated boundary-layer conditions. Such sustained humidity suppresses evaporative cooling from the body surface of dairy cattle and reduces their capacity to dissipate metabolic heat, thereby amplifying the physiological impact of rising air temperatures and extending the duration of heat-stress exposure [30,31].

Changes in precipitation patterns (Figure 3c) have further modified the thermal environment across Jiangsu Province. A clear southward intensification of rainfall—+20 to +40 mm per decade in the south versus nearly stable or slightly declining trends in the north—indicates a more concentrated monsoon season and stronger seasonal contrasts. This contrast is particularly pronounced in southern Jiangsu, where increases are both stronger in magnitude and spatially consistent, with large contiguous areas exhibiting statistically significant upward trends, while northern regions show more heterogeneous patterns, including localized but significant declines, especially in the northwestern part of the province. Although mean relative humidity has slightly decreased overall, frequent rainfall events and persistent soil moisture in the southern plain still create periods of high near-surface humidity during summer, which hinder evaporative heat dissipation in dairy cattle and sustain elevated THI levels. Wind speed (Figure 3d) has also declined consistently across the province, with reductions of 0.1–0.4 m/s per decade. This long-term weakening of near-surface wind limits natural ventilation in open or semi-open barns, thereby reducing the efficiency of convective and evaporative heat loss from cattle during hot and humid conditions. Overall, the reduction in air movement narrows the thermal comfort range for dairy cattle, amplifying the impact of high-temperature episodes while allowing only modest relief from cold extremes.

Collectively, the observed climatic evolution indicates a transition toward a warmer, wetter, and less ventilated climate system, which is particularly evident in the southern and coastal regions of Jiangsu. The combined effects of sustained warming, more concentrated rainfall, and reduced wind speed have led to a steady increase in the frequency, intensity, and duration of heat-stress conditions for dairy cattle. Meanwhile, despite the overall warming trend, the persistence of strong winter monsoon intrusions continues to cause cold-stress risks in the northern plain, although their severity has gradually declined. The coexistence of these opposing thermal extremes defines Jiangsu’s distinctive dual-stress climatic pattern, shaping both the temporal variability and spatial redistribution of livestock suitability across the province.

### 3.2. Spatiotemporal Evolution of Heat- and Cold-Stress Days in Jiangsu

**Figure 4:**
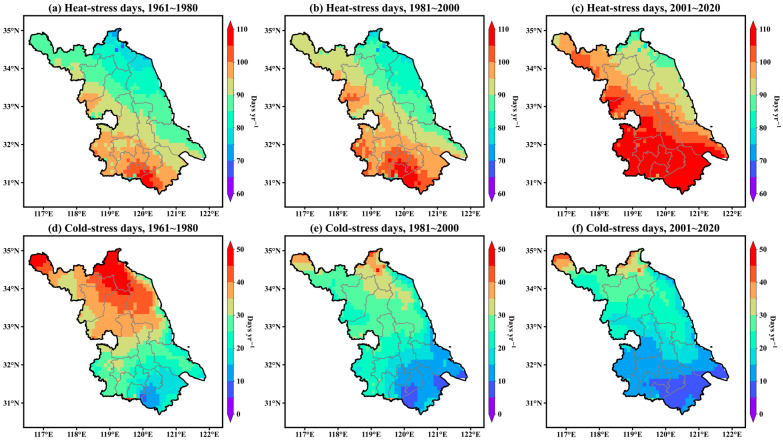
Spatial distribution of annual heat-stress days (**a**–**c**) and cold-stress days (**d**–**f**) for three climatic periods (1961–1980, 1981–2000, and 2001–2020) across Jiangsu Province.

To examine the long-term evolution of thermal stress days across Jiangsu Province, Figure 4 illustrates the spatial distribution of annual heat-stress and cold-stress days for three climatic periods (1961–1980, 1981–2000, and 2001–2020). Together, the two-map series depict substantial reorganization of the provincial thermal environment over the past six decades.

The upper row (heat-stress days) reveals a stepwise intensification and northward expansion of heat-stress conditions. During 1961–1980, days above the heat-stress threshold were largely confined to the lower Yangtze and Taihu regions, typically ranging from 90 to 105 days per year, with the Huai River marking a clear climatic transition to fewer events. By 1981–2000, a contiguous core of higher frequencies (95–110 days) emerged in the southern and central prefectures, and the 70-day isoline advanced into the mid-province. In 2001–2020, the pattern evolved into a province-wide distribution: southern and coastal counties frequently exceeded 105 days, central Jiangsu entered the 100–105-day class, and even the northern plain reached 90–95 days. This northward migration of frequent heat-stress occurrence aligns with the climatic shifts documented in Section 3.1—namely sustained warming, high summer humidity, and reduced wind speed that limits convective and evaporative cooling. The coherent progression across decades indicates not merely intensified summer warming, but an extended duration of the THI surpassing the threshold—with earlier spring onset and/or delayed autumn retreat—which explains the cumulative increase in annual heat-stress days without the need for more extreme temperature anomalies.

The lower row (cold-stress days) presents the opposite pattern: a systematic contraction of wintertime cold stress from a broad area in 1961–1980 (northern prefectures commonly experiencing 35–50 days, with much lower values in the south) to a narrower zone in 1981–2000, and finally to 2001–2020, when most of southern and central Jiangsu fell into the ≤10–15-day category, while the northern cities retained a limited residual zone of ≈15–25 days. This decline corresponds closely with the warming trend and the reduction in near-surface wind speeds described earlier. Nevertheless, the persistence of a core region of cold-stress days in the northwestern interior suggests that winter monsoon incursions remain active. Although the overall severity of cold stress has diminished, its continued presence in northern Jiangsu sustains a provincial-scale dual-stress pattern, characterized by an increasing heat-stress burden in the south and coast and residual cold-stress exposure in the north.

Taken together, these trends suggest a northward shift in the climatic suitability for dairy farming. Areas that were previously thermally favorable in summer are now frequently pushed beyond the THI threshold of 72, while regions that were once constrained by low winter temperatures have become less restrictive—but not uniformly so. The overall outcome is a south-to-north redistribution of thermally optimal conditions, with increasing heat-stress pressure on intensive dairy systems in the south (more days demanding cooling, ventilation, and water use) and continuing requirements for cold protection measures (wind barriers, insulation, and nutritional adjustment) in the northern plain. These findings highlight the need to strengthen regional forecasting and early-warning systems for both heat and cold stress, enabling adaptive management and improved resilience of dairy production under ongoing climate change.

### 3.3. Provincial-Level Distribution of THI-Suitable Days for Dairy Cattle

**Figure 5:**
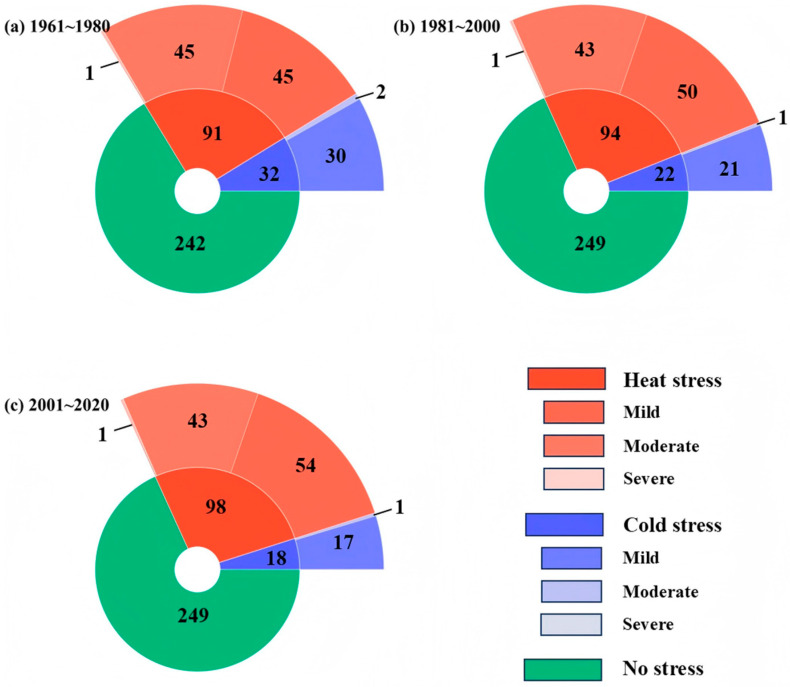
Temporal changes in the annual number of thermal stress and no-stress days for dairy cattle in Jiangsu Province during three climatic periods: (**a**) 1961–1980, (**b**) 1981–2000, and (**c**) 2001–2020.

To assess long-term changes in thermal suitability for dairy cattle across Jiangsu Province, Figure 5 summarizes the proportions of no-stress, heat-stress, and cold-stress days for three climatic periods (1961–1980, 1981–2000, and 2001–2020). The results provide a clear depiction of how warming and seasonal asymmetry have reshaped the comfort environment for dairy farming over the past six decades.

During 1961–1980, no-stress conditions (38 < THI ≤ 72) prevailed for most of the year, averaging 242 days, or approximately 66% of the annual cycle. Heat-stress days were frequent, averaging 91 days yr^−1^, while cold-stress days occurred on about 32 days yr^−1^, indicating that heat stress was the dominant climatic constraint for dairy cattle in the early decades. The prevalence of mild to moderate heat stress during this period reflects the combined influence of relatively high summer temperatures and strong solar radiation, while higher wind speeds and shorter duration of cold events limited the impact of winter cold stress. Between 1981 and 2000, the number of no-stress days increased slightly to 249 days yr^−1^, primarily due to a reduction in cold-stress events (from 32 to 22 days yr^−1^). However, this apparent improvement in annual thermal comfort was accompanied by a modest rise in heat-stress frequency. This period marks a transitional phase in which the dominance of cold stress began to weaken and the influence of heat stress became increasingly significant—consistent with the warming trend and reduced wind ventilation described in Section 3.1. In 2001–2020, the thermal environment underwent a marked transformation. The number of heat-stress days increased substantially compared with the early period, rising from 91 to 98 days yr^−1^, while cold-stress days declined sharply to 18 days yr^−1^. Although the total number of no-stress days remained relatively stable (249 days yr^−1^), the seasonal distribution shifted sharply: summer heat stress intensified and lengthened, while winter cold stress contracted but did not disappear entirely. The persistence of mild to moderate cold stress, especially in northern areas, indicates that winter remains a non-negligible physiological challenge for cattle, even under a warming climate.

Taken as a whole, the records point to a strengthening heat-dominated regime. From 1961–1980 to 2001–2020, heat-stress days rose from 91 to 98 days yr^−1^, while cold-stress days declined from 32 to 18 days yr^−1^, with no-stress days remaining near 240–250 days yr^−1^. This evolution is consistent with sustained warming and a weakening of near-surface wind, which together increase the likelihood and persistence of THI > 72 events, while simultaneously reducing the frequency of THI ≤ 38 days. The net effect is a narrower effective comfort window driven mainly by longer and more frequent summer heat episodes, alongside a shorter—but still relevant—winter cold-stress season, especially in northern Jiangsu. From a management standpoint, southern and coastal dairies face increasing pressure to mitigate heat (enhanced ventilation, shading, evaporative cooling, and reliable water supply), whereas northern herds still require targeted winter protection (insulation, wind barriers, and nutritional adjustment) to handle intermittent cold. The persistence of roughly one-third of the year with some form of thermal stress underscores the need for integrated forecasting and early-warning systems for both heat and cold stress, enabling adaptive, region-specific management under ongoing climate change.

### 3.4. Suitable Days for Dairy Cattle Across Different Cities in Jiangsu

Table 2 summarizes the multi-decadal evolution of cold-stress (CS), heat-stress (HS), and no-stress (NS) days across 13 prefecture-level cities in Jiangsu Province for three climatic periods (1961–1980, 1981–2000, and 2001–2020). The data reveal consistent spatial gradients and temporal transitions that collectively demonstrate how regional climate change has reshaped the thermal suitability for dairy farming across the province.

A coherent pattern of declining cold-stress frequency and increasing heat-stress occurrence is observed across nearly all cities. From 1961–1980 to 1981–2000, cold-stress days decreased by 20–40% in most southern and central cities, with Suzhou and Wuxi exhibiting the most pronounced reductions (−45% and −35%, respectively). This decline continued during 2001–2020, with several southern cities—such as Suzhou, Changzhou, and Nanjing—experiencing fewer than 15 cold-stress days per year, underscoring the diminishing influence of winter cold surges in these lower-latitude, low-altitude areas. In contrast, northern cities such as Xuzhou, Lianyungang, and Suqian still recorded around 25–30 cold-stress days in the most recent period, highlighting the persistent influence of continental air masses and the residual strength of the East Asian winter monsoon.

The trajectory of heat-stress days shows the opposite trend. Compared with 1961–1980, the period 1981–2000 saw moderate increases (≈+3 to +6 days yr^−1^) across most locations, while the acceleration during 2001–2020 was substantial—up to +14 to +17 days yr^−1^ in the central and southern urban clusters (Nanjing, Wuxi, Changzhou, Suzhou). These regions, situated in the lower Yangtze River corridor, have undergone rapid urbanization and industrialization, intensifying surface heat retention and humidity, both of which contribute to higher THI values. The persistence of elevated humidity during monsoon months and the reduction in wind speed further compound the heat load, explaining why southern Jiangsu now experiences over 100 heat-stress days per year, compared with 80–90 days in the 1960s–1970s.

The number of no-stress days (thermal comfort) remained relatively stable at the provincial scale but displayed divergent tendencies among cities. Southern and coastal cities such as Nantong, Suzhou, and Yancheng gained approximately +5 to +9 no-stress days between 1961–1980 and 1981–2000, largely due to milder winters, but subsequently experienced slight declines (−4 to −12 days) in 2001–2020 as prolonged heat events eroded the comfort window. Conversely, northern and inland areas (e.g., Xuzhou, Lianyungang, Suqian) recorded minor improvements in no-stress days during the middle period, followed by renewed decreases in the latest decades, indicating that the northern plain is gradually transitioning from cold-limited to heat-limited conditions.

Overall, the data reveal a south-to-north shift in climatic constraints on dairy production. In early decades, thermal comfort was curtailed primarily by winter cold, particularly in northern Jiangsu. In recent decades, it is increasingly limited by prolonged summer heat in the south. This evolving spatial asymmetry produces a dual-stress pattern—intensified heat exposure in the southern half of the province and persistent, though weakening, cold stress in the north. Such heterogeneity underscores the importance of region-specific adaptation strategies: southern farms should prioritize ventilation, shading, and evaporative cooling systems to mitigate rising heat stress, whereas northern operations must maintain adequate winter insulation and cold protection. Given the pronounced increase in heat-stress frequency and the spatial redistribution of thermal stress documented above, there is a clear need for operational tools that can support near-term decision-making in dairy management.

### 3.5. Application of the Forecasted Platform in Jiangsu Province

#### 3.5.1. Short-Term Forecasting of Thermal Stress and Associated Meteorological Conditions

**Figure 6:**
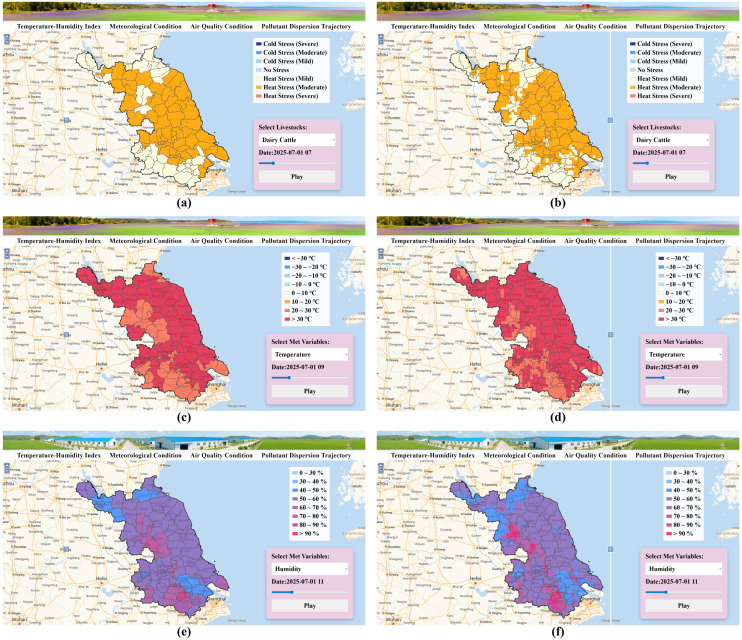
Forecasted thermal stress and meteorological conditions for the next 48 h across Jiangsu Province, generated by the WRF-based Jiangsu Dairy Thermal Stress Forecast Platform. (**a**) County-level thermal stress distribution; (**b**) Grid-level thermal stress distribution; (**c**) County-level temperature distribution; (**d**) Grid-level temperature distribution; (**e**) County-level humidity distribution; (**f**) Grid-level humidity distribution.

The spatial redistribution and intensification of thermal stress highlighted in Section 3.1, Section 3.2, Section 3.3 and Section 3.4 underscore the increasing challenges posed by the rising frequency of heat-stress days. To support proactive decision-making in response to these emerging climatic risks, we developed a forecasting platform that provides short-term predictions of thermal stress and associated environmental conditions. The platform integrates outputs from the WRF model to produce 48 h forecasts of temperature, relative humidity, wind speed, and derived THI values at 9 km resolution. The results are rendered through an OpenLayers-based interface that allows users to dynamically switch between grid-scale and administrative (county-level) visualizations (Figure 6). To ensure the reliability of the forecasting system, the performance of the WRF model was quantitatively evaluated against observations aggregated at the city level across 13 prefecture-level cities in Jiangsu Province (Figure S1). The evaluation covered both summer (1 July–31 August 2025) and winter (1 November–31 December 2025) periods, and included air temperature, relative humidity, and derived THI. The results demonstrate strong correlations and moderate errors across all variables, supporting the capability of the model to reliably reproduce meteorological conditions and associated thermal stress patterns (Tables S1–S6).

The forecast maps for THI, temperature, and relative humidity clearly reveal the coupled evolution of meteorological drivers behind dairy thermal stress. At the initialization stage (Figure 6a,b), most regions of Jiangsu exhibit conditions classified as no stress or mild heat stress, consistent with moderate early-morning temperatures and relatively low humidity. As forecast time advances (Figure 6c,d), diurnal heating intensifies across the central and southern plains, with temperature gradients exceeding 30 °C and THI values rising rapidly above 72. This transition reflects the strong sensitivity of the THI to temperature amplification under moist atmospheric conditions. Meanwhile, relative humidity fields (Figure 6e,f) display a progressive decline in the northwest, contrasting with moisture accumulation in the coastal and Taihu regions, where the combination of high humidity (>70%) and weak ventilation sustains elevated THI values into the evening hours.

The joint visualization of these variables illustrates the tight meteorological coupling underlying heat-stress formation: thermal load is not determined by temperature alone, but by the concurrent variation of humidity, wind speed, and boundary-layer stability. In particular, the platform’s spatially explicit forecasts enable detection of short-lived but spatially extensive stress events—such as humid-heat episodes following overnight stagnation—which are often underestimated by station-based analyses. The grid-level display provides high spatial granularity for regional risk mapping, whereas the county-level aggregation supports managerial decision-making and adaptive scheduling of cooling and feeding operations.

Through the integration of dynamic meteorological simulation and THI-based classification, the forecasting module bridges climatological insight with actionable prediction. This capability establishes a technical foundation for early warning of dairy thermal stress in Jiangsu, offering both scientific and practical value for climate adaptation, farm-level management, and regional livestock planning.

#### 3.5.2. Atmospheric Transport and Air-Quality Conditions Relevant to Dairy Production Environments

To extend the thermal stress forecasts and provide a more complete characterization of the atmospheric environment relevant to dairy production, the platform incorporates a STILT-based forward dispersion module and a CMAQ-driven air-quality prediction system. This integrated design reflects the fact that the conditions experienced by dairy cattle are shaped not only by temperature and humidity but also by the movement of air masses and the composition of the surrounding atmosphere.

**Figure 7:**
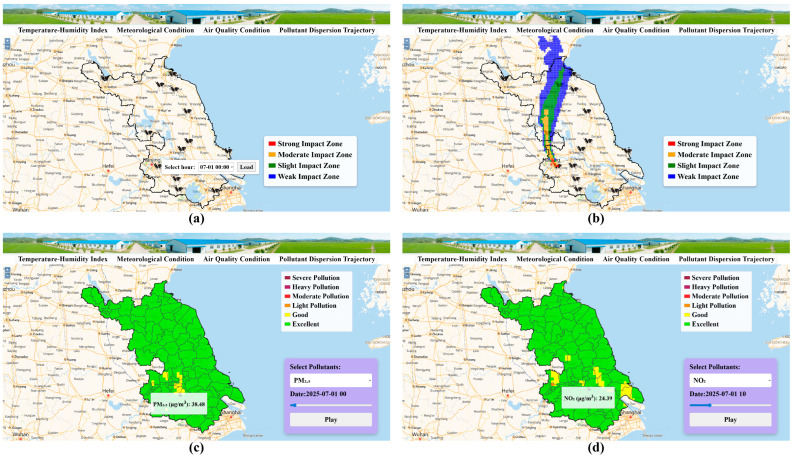
Visualization of pollutant-dispersion trajectories and air-quality conditions generated by the WRF-STILT and WRF-CMAQ integrated module of the Jiangsu Dairy Environmental Forecast Platform. (**a**) Interface for selecting the simulation time; (**b**) Simulated pollutant dispersion trajectory based on the STILT model; (**c**) Spatial distribution of PM_2.5_ concentration; (**d**) Spatial distribution of NO_2_ concentration.

The STILT forward trajectory module simulates the pathways of air parcels originating from dairy production locations and projects their movement over the subsequent 24 h. As shown in Figure 7a,b, the system allows users to specify a forecast hour within the 48 h prediction window and visualize the corresponding ensemble of forward trajectories. The resulting footprints—classified into strong, moderate, slight, and weak impact zones—highlight the spatial extent over which air masses disperse and interact with the surrounding environment. The air-quality fields displayed in Figure 7c,d are generated by the CMAQ model, dynamically coupled with the same WRF meteorology used for THI forecasting. The predicted spatial distributions of PM_2.5_ and NO_2_ indicate that most areas of Jiangsu maintain “good” to “excellent” air-quality levels during the forecast period, with only limited pockets of moderate pollution. Although these pollutants do not directly enter the THI calculation, their physiological impacts may compound heat stress—reducing respiratory efficiency, increasing oxidative load, and elevating susceptibility in vulnerable cattle groups—thus representing an additional atmospheric stress pathway. By combining forward trajectories with CMAQ-derived pollutant forecasts, the platform provides a unified environment for interpreting both local meteorological drivers and regional atmospheric transport influences. This coupled approach enhances early-warning capability, helping producers anticipate not only when thermal stress will occur, but also whether atmospheric stagnation, pollutant buildup, or long-range transport may intensify environmental risks. For dairy-farm management, such integrated information supports proactive decisions on ventilation operation, timing of outdoor exposure, and protection of sensitive animals during periods when meteorological and air-quality factors jointly elevate physiological load.

Overall, the pollutant-dispersion and air-quality modules extend the scope of the forecasting platform beyond temperature and humidity alone, offering a more comprehensive basis for environmental risk assessment and adaptive management under increasingly variable atmosphere–climate conditions.

## 4. Discussion

### 4.1. Implications for Farm Practices and Regional Policy

The analyses presented in Section 3.1, Section 3.2, Section 3.3 and Section 3.4 demonstrate a coherent shift in the thermal environment of Jiangsu Province, characterized by a province-wide intensification and northward expansion of heat-stress days alongside a steady, though incomplete, contraction of cold-stress conditions. This evolving dual-season pattern provides a critical context for understanding climate-related risks to dairy production in the region. Heat-stress exposure has increased most rapidly in southern and coastal prefectures, whereas northern areas continue to experience a non-negligible period of cold stress. City-level comparisons further confirm this redistribution, with most prefectures showing more frequent warm-season stress and spatially heterogeneous reductions in winter cold exposure.

These changes indicate that dairy cattle in Jiangsu are increasingly subjected to two distinct but interacting physiological pressures: a longer and more intense warm-season stress period, driven by rising temperatures, persistently high summer humidity, and reduced wind ventilation, and a shortened yet persistent winter stress window, particularly in the northern plain where cold air incursions remain climatologically active. The coexistence of these seasonal stressors implies higher cumulative metabolic demands and narrower thermal comfort margins for dairy cattle throughout the year. From a farm-management perspective, the documented increase in THI exceedance suggests that conventional ventilation and shading practices may no longer be sufficient in many parts of the province. In southern and central prefectures, where heat-stress thresholds are now exceeded for approximately 80–110 days per year, enhanced cooling strategies—such as high-capacity ventilation, evaporative cooling systems, and automated climate-control technologies—are likely to become essential for maintaining milk yield, reproductive performance, and animal welfare [32,33,34]. Nutritional adjustments, including electrolyte supplementation, increased dietary energy density, and antioxidant support, may further help alleviate heat-induced metabolic strain [35,36]. At the same time, the persistence of cold-stress days in northern Jiangsu (approximately 15–25 days yr^−1^ during 2001–2020) indicates that winter resilience remains an important consideration. Although the overall severity of cold stress has declined, vulnerable groups such as calves, transition cows, and high-producing animals may still require effective insulation, draft control, and seasonally adjusted feeding strategies.

At the regional policy level, these findings highlight the importance of climate-informed planning for dairy development. Prefectures experiencing rapid intensification of heat stress, including Suzhou, Wuxi, Changzhou, and Nantong, would benefit from prioritizing investment in heat-resilient barn design, water-efficient cooling technologies, and technical support for precision environmental control [37,38]. In contrast, northern prefectures such as Xuzhou, Lianyungang, and Suqian should continue to receive targeted support for winterization measures and early-warning systems capable of responding to episodic cold surges. More broadly, the spatiotemporal redistribution of climatic suitability identified in Section 3.3 suggests that future dairy expansion, farm siting, and zoning regulations should explicitly incorporate thermal stress projections rather than relying solely on historical climate conditions.

Overall, the emerging dual-stress pattern documented in this study underscores the need to move beyond uniform management approaches toward region-specific and season-specific adaptation strategies. Such a combined intensification of humid-heat stress and residual winter cold is not unique to Jiangsu but reflects challenges increasingly faced by dairy systems in many warming, moisture-rich regions worldwide. Aligning farm-level practices and regional policy interventions with observed and projected climatic trajectories will be critical for sustaining dairy productivity, animal welfare, and system resilience in Jiangsu Province under continued climate warming.

### 4.2. Integrating Forecasting Tools into Climate-Resilient Dairy Production Systems

The development of the WRF-STILT forecasting platform (Section 2.4) and its application results (Section 3.5) extend the historical analyses of this study into a practical framework for short-term assessment and anticipatory adaptation. The platform directly addresses the key climatic challenges identified in earlier sections, including the increasing frequency of heat-stress events, pronounced spatial heterogeneity in thermal risk, and the role of atmospheric circulation in modulating local stress conditions.

The THI forecasts presented in Section 3.5 demonstrate how short-term variations in temperature, humidity, and airflow can rapidly elevate thermal stress levels, consistent with the long-term intensification of summer heat stress documented in Section 3.2. The simultaneous visualization of grid-scale and county-level THI patterns reflects the spatial variability in thermal exposure identified across Jiangsu Province, particularly along coastal–inland and north–south gradients. By providing advance information on periods when the THI is likely to exceed critical thresholds, the platform translates climatological mechanisms into an operational context and supports short-term preparedness at both farm and regional scales. The incorporation of atmospheric transport and air-quality modules further complements the thermal stress assessment. Although air-quality variables are not included in the THI calculation, STILT-based trajectory analyses reveal airflow pathways and stagnation patterns that are consistent with the observed long-term decline in near-surface wind speeds (Section 3.1). Periods of weak ventilation and synoptic stagnation are often associated with both elevated THI and pollutant accumulation, suggesting that multiple environmental stressors may co-occur under specific meteorological conditions [39,40]. Visualizing these components together provides a more integrated depiction of the atmospheric environment surrounding dairy production systems.

From an adaptation perspective, forecasting tools offer several important advantages [41]. At the farm level, advance identification of high-THI periods enables a shift from reactive to anticipatory management, including adjustment of ventilation and cooling strategies, modification of feeding schedules, and precautionary measures for vulnerable animals during predicted stress windows. At the regional level, the platform provides a basis for early-warning services, allowing agricultural agencies to disseminate targeted advisories for specific prefectures or production clusters. In addition, standardized forecasts of thermal stress create an objective reference for climate-smart policy design, including heat-stress response plans, infrastructure investment strategies, and the evaluation of environmental conditions associated with new or expanded dairy facilities. Looking forward, integrating forecast platforms with on-farm sensor networks, automated barn monitoring, and machine-learning-based stress models could further strengthen Jiangsu’s adaptive capacity [42,43]. The synergy between historical trend analysis and real-time forecasting underscores the critical role of digital tools in shaping a climate-resilient dairy production system. Importantly, this forecast-to-decision framework provides a transferable climate-service template that can be adapted to other dairy-producing regions worldwide facing increasing heat-stress risk under climate warming.

## 5. Conclusions

This study quantified changes in thermal stress conditions for dairy cattle in Jiangsu Province over the period 1961–2020 and assessed their implications using both historical analysis and short-term forecasting. The results show a clear and consistent increase in heat-stress exposure across the province. On average, the annual number of heat-stress days (THI > 72) increased by approximately 10–20 days between the early period (1961–1980) and the most recent decades (2001–2020), with southern and coastal prefectures frequently experiencing more than 100 heat-stress days per year. At the same time, heat-stress conditions expanded northward, and areas that previously experienced moderate summer stress now regularly exceed heat-stress thresholds. In contrast, cold-stress exposure (THI ≤ 38) declined over the same period but did not disappear. Cold-stress days decreased substantially in southern and central Jiangsu, falling to fewer than 10–15 days per year in many locations by 2001–2020. However, northern prefectures still experienced approximately 15–25 cold-stress days annually, indicating that winter cold remains a relevant constraint for dairy cattle in these areas. Together, these trends demonstrate a redistribution of thermal stress characterized by intensified and prolonged summer heat stress in the south and a persistent, though reduced, winter cold-stress window in the north. The combined changes in heat-stress and cold-stress days resulted in a spatial shift in climatic suitability for dairy production, while the total number of thermally comfortable days remained relatively stable at the provincial scale.

By linking long-term climate analysis with short-term THI forecasting, the WRF-STILT-based platform developed in this study provides a practical means to assess when and where thermal stress is likely to occur. The platform translates observed climatic trends into short-term forecasts of heat and cold stress and associated atmospheric conditions, supporting early identification of stress events at both grid and administrative scales. The findings indicate that sustaining dairy production in Jiangsu under continued climate warming will require explicit consideration of changing thermal stress durations rather than reliance on historical climate norms. Quantifying the evolution of thermal stress days, together with targeted short-term forecasting, offers a concrete basis for region-specific adaptation strategies in dairy management, while also providing a transferable reference for climate-resilient dairy systems in other warming and humid regions worldwide.

## Figures and Tables

**Table 1:** Temperature–humidity index (THI) thresholds used to characterize heat-stress, cold-stress, and no-stress conditions for dairy cattle.

Stress Level	Heat Stress	Cold Stress
Comfort (no stress)	THI ≤ 72	THI > 38
Mild	72 < THI ≤ 78	25 < THI ≤ 38
Moderate	78 < THI ≤ 88	8 < THI ≤ 25
Severe	THI > 88	THI ≤ 8

**Table 2:** Number of cold-stress (CS), heat-stress (HS), and no-stress (NS) days in different cities of Jiangsu Province during three climatic periods (1961–1980, 1981–2000, and 2001–2020).

City	1961–1980			1981–2000			2001–2020		
	CS	HS	NS	CS	HS	NS	CS	HS	NS
Nanjing	28	96	241	23	97	245	13	112	240
Wuxi	20	98	247	13	103	249	11	114	240
Xuzhou	41	88	236	29	92	244	29	99	237
Changzhou	24	98	243	17	101	247	10	115	240
Suzhou	18	98	249	10	101	254	8	115	242
Nantong	24	90	251	14	91	260	13	102	250
Lianyungang	50	78	237	34	83	248	30	89	246
Huai’an	40	88	237	28	92	245	22	98	245
Yancheng	37	82	246	26	84	255	21	94	250
Yangzhou	34	91	240	25	93	247	16	103	246
Zhenjiang	28	94	243	21	97	247	12	109	244
Taizhou	30	90	245	22	92	251	17	103	245
Suqian	42	88	235	27	93	245	24	98	243

## Data Availability

The raw data supporting the conclusions of this article will be made available by the authors on request. The demo of the platform was deployed on https://animal-thi.github.io/.

## Supplementary Materials

The following supporting information can be downloaded at https://www.mdpi.com/article/10.3390/ani16081166/s1. Figure S1. Meteorological monitoring sites in Jiangsu Province. Table S1. Statistical evaluation of WRF-simulated air temperature compared with observations across Jiangsu Province during the summer period (1 July−31 August 2025). Table S2. Statistical evaluation of WRF-simulated relative humidity compared with observations across Jiangsu Province during the summer period (1 July−31 August 2025). Table S3. Statistical evaluation of the WRF-simulated temperature–humidity index compared with observations across Jiangsu Province during the summer period (1 July−31 August 2025). Table S4. Statistical evaluation of WRF-simulated air temperature compared with observations across Jiangsu Province during the winter period (1 November−31 December 2025). Table S5. Statistical evaluation of WRF-simulated relative humidity compared with observations across Jiangsu Province during the winter period (1 November−31 December 2025). Table S6. Statistical evaluation of the WRF-simulated temperature–humidity index compared with observations across Jiangsu Province during the winter period (1 November−31 December 2025).
