# Supplementary material for: Climate Change Reshapes Thermal Suitability for Dairy Cattle in Jiangsu Province (1961–2020)
Source: Animals (Basel). 2026 Apr 10;16(8):1166. doi: 10.3390/ani16081166 (PMC13113301; doi:10.3390/ani16081166)
Supplement: Supplementary file 1 [file animals-16-01166-s001.zip › animals-4163583-supplementary.pdf]

Supply materials for

Climate change reshapes thermal suitability for dairy cattle in Jiangsu Province (1961–2020)

Guangyi Yang[1, 2, 3], Fei Liu[1], Guangqin Zhu[1, *], Qiong Liu[1], Chao Wang[1], Dong Li[1], Zhongrui Guo[1] and Hongmei Zhao[2, *]

[1] School of Ecology and Environment, Xuzhou Vocational College of Bioengineering, Xuzhou 221006, China
[2] Northeast Institute of Geography and Agroecology, Chinese Academy of Sciences, Changchun, 130102, China
[3] University of Chinese Academy of Sciences, Beijing, 100049, China
* Correspondence: qin823727@163.com and zhaohongmei@iga.ac.cn.

Section S1. Validation of the WRF-based forecasting system

To evaluate the performance of the WRF-based forecasting system, simulated meteorological variables (air temperature, relative humidity, and derived THI) were quantitatively compared with observations from 13 cities across Jiangsu Province for both summer (1 July–31 August 2025) and winter (1 November–31 December 2025) periods (Tables S1–S6).

For air temperature, the model shows strong agreement with observations in both seasons. During summer (Table S1), correlation coefficients (R) range from 0.87 to 0.92, indicating a high level of temporal consistency, although a systematic negative bias (−1.29 to −2.01 °C) suggests a slight underestimation of temperature. RMSE and MAE values remain moderate (2.10−2.73 °C and 1.83−2.36 °C, respectively), indicating acceptable simulation accuracy. In winter (Table S4), model performance improves further, with higher correlations (R = 0.96−0.97), reduced RMSE (1.29−2.08 °C), and a small positive bias (0.26−1.44 °C), suggesting a slight overestimation but overall improved stability under cooler conditions. Relative humidity simulations exhibit slightly lower but still satisfactory performance. In summer (Table S2), correlations range from 0.85 to 0.91, with a consistent positive bias (approximately +3.84% to +9.50%), indicating that the model tends to overestimate humidity under warm and moist conditions. RMSE values range from 7.74% to 13.48%. In winter (Table S5), correlations remain comparable (R = 0.82−0.92), but the bias becomes predominantly negative (−0.99% to −11.15%), suggesting an underestimation of humidity in colder conditions. These seasonal differences likely reflect challenges in representing boundary-layer moisture processes and cloud–radiation interactions. For the temperature–humidity index (THI), which integrates both temperature and humidity, the model maintains good performance despite the propagation of uncertainties from both variables. In summer (Table S3), correlations range from 0.80 to 0.88, with a slight negative bias (−1.64 to −2.39), indicating a tendency to underestimate thermal stress intensity. RMSE values range from 2.37 to 3.21. In winter (Table S6), correlations improve

significantly (R = 0.93–0.96), with a modest positive bias (0.33–2.74) and RMSE values between 2.27 and 4.30.

Overall, the WRF-based forecasting system demonstrates reliable performance in reproducing both meteorological variables and derived THI across different seasons and regions. Although some systematic biases exist, their magnitude remains moderate and does not affect the ability of the model to capture temporal variability and spatial patterns of thermal stress. These results provide strong support for the application of the forecasting platform in short-term thermal-stress assessment and early warning for dairy cattle in Jiangsu Province.

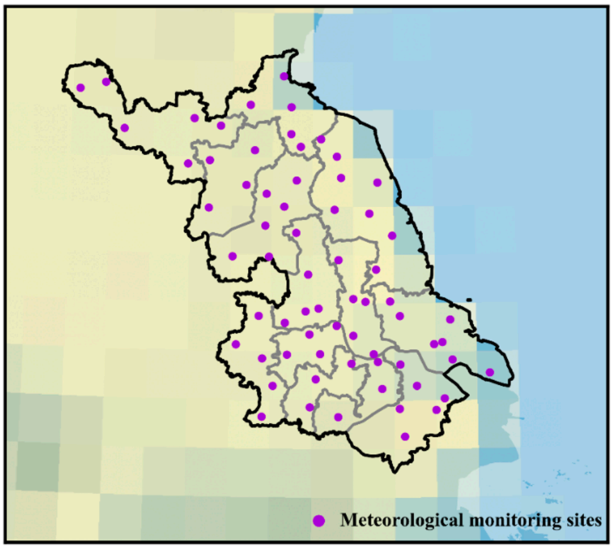

Figure S1. Meteorological monitoring sites in Jiangsu Province

Table S1. Statistical evaluation of WRF-simulated air temperature compared with observations across Jiangsu Province during the summer period (1 July–31 August 2025)

City	N (hour)	R	Bias	RMSE	MAE
Nanjing	1488	0.92	-1.75	2.39	2.07
Wuxi	1488	0.89	-1.76	2.29	1.97
Xuzhou	1488	0.92	-1.29	2.10	1.83
Changzhou	1488	0.89	-1.83	2.67	2.28
Suzhou	1488	0.87	-1.83	2.73	2.36
Nantong	1488	0.91	-1.78	2.29	1.99
Lianyungang	1488	0.90	-1.80	2.48	2.16
Huai'an	1488	0.91	-1.58	2.25	1.91
Yancheng	1488	0.92	-1.70	2.28	1.95
Yangzhou	1488	0.91	-1.81	2.47	2.09
Zhenjiang	1488	0.92	-1.97	2.42	2.11
Taizhou	1488	0.91	-2.01	2.59	2.23
Suqian	1488	0.89	-1.68	2.46	2.08

Table S2. Statistical evaluation of WRF-simulated relative humidity compared with observations across Jiangsu Province during the summer period (1 July–31 August 2025)

City	N (hour)	R	Bias	RMSE	MAE
Nanjing	1488	0.88	7.83	11.61	9.52
Wuxi	1488	0.85	9.50	12.57	10.33
Xuzhou	1488	0.91	4.59	9.09	7.62
Changzhou	1488	0.85	8.28	13.40	11.26
Suzhou	1488	0.85	7.93	13.48	11.42
Nantong	1488	0.90	7.26	9.87	8.44
Lianyungang	1488	0.91	6.48	9.73	8.28
Huai'an	1488	0.90	3.84	7.74	6.18
Yancheng	1488	0.91	4.12	7.77	6.31
Yangzhou	1488	0.87	6.23	10.62	8.33
Zhenjiang	1488	0.85	9.48	12.73	10.38
Taizhou	1488	0.88	9.15	12.55	10.75
Suqian	1488	0.88	5.52	9.83	7.87

Table S3. Statistical evaluation of WRF-simulated temperature-humidity index compared with observations across Jiangsu Province during the summer period (1 July–31 August 2025).

City	N (hour)	R	Bias	RMSE	MAE
Nanjing	1488	0.84	-1.89	2.84	2.31
Wuxi	1488	0.80	-1.64	2.37	1.97
Xuzhou	1488	0.88	-1.65	2.53	2.07
Changzhou	1488	0.81	-2.04	2.96	2.40
Suzhou	1488	0.80	-2.11	2.92	2.40
Nantong	1488	0.84	-2.06	2.79	2.30
Lianyungang	1488	0.82	-2.23	3.19	2.63
Huai'an	1488	0.84	-2.21	3.13	2.56
Yancheng	1488	0.85	-2.39	3.21	2.65
Yangzhou	1488	0.85	-2.20	3.06	2.52
Zhenjiang	1488	0.84	-1.95	2.64	2.23
Taizhou	1488	0.85	-2.17	2.99	2.44
Suqian	1488	0.83	-2.10	3.05	2.48

Table S4. Statistical evaluation of WRF-simulated air temperature compared with observations across Jiangsu Province during the winter period (1 November–31 December 2025)

City	N (hour)	R	Bias	RMSE	MAE
Nanjing	1464	0.97	1.12	1.79	1.47
Wuxi	1464	0.96	0.76	1.76	1.29
Xuzhou	1464	0.96	1.09	1.88	1.53
Changzhou	1464	0.97	0.55	1.39	1.14
Suzhou	1464	0.97	0.26	1.29	1.04
Nantong	1464	0.97	0.97	1.66	1.28
Lianyungang	1464	0.97	0.90	1.61	1.33
Huai'an	1464	0.97	1.30	1.94	1.59
Yancheng	1464	0.97	1.08	1.76	1.41
Yangzhou	1464	0.97	1.44	2.08	1.67
Zhenjiang	1464	0.97	0.50	1.33	1.04
Taizhou	1464	0.97	0.76	1.58	1.22
Suqian	1464	0.96	0.88	1.79	1.44

Table S5. Statistical evaluation of WRF-simulated relative humidity compared with observations across Jiangsu Province during the winter period (1 November–31 December 2025)

City	N (hour)	R	Bias	RMSE	MAE
Nanjing	1464	0.86	-6.10	13.95	10.52
Wuxi	1464	0.82	-1.03	12.18	8.64
Xuzhou	1464	0.85	-11.15	16.73	12.76
Changzhou	1464	0.87	-4.13	13.26	10.21
Suzhou	1464	0.90	-0.99	10.94	8.00
Nantong	1464	0.92	-1.77	9.49	6.93
Lianyungang	1464	0.90	-5.47	11.43	8.67
Huai'an	1464	0.87	-9.49	14.96	11.44
Yancheng	1464	0.88	-5.54	12.51	9.24
Yangzhou	1464	0.85	-9.08	15.80	11.71
Zhenjiang	1464	0.87	-2.72	11.29	8.09
Taizhou	1464	0.89	-3.20	11.65	8.85
Suqian	1464	0.86	-8.58	15.01	11.42

Table S6. Statistical evaluation of WRF-simulated temperature-humidity index compared with observations across Jiangsu Province during the winter period (1 November–31 December 2025)

City	N (hour)	R	Bias	RMSE	MAE
Nanjing	1464	0.94	1.96	3.51	2.64
Wuxi	1464	0.93	1.23	3.38	2.32
Xuzhou	1464	0.94	2.47	3.98	3.04
Changzhou	1464	0.95	0.87	2.51	2.00
Suzhou	1464	0.96	0.33	2.27	1.77
Nantong	1464	0.96	1.54	2.93	2.09
Lianyungang	1464	0.96	1.68	3.01	2.35
Huai'an	1464	0.94	2.48	3.88	2.92
Yancheng	1464	0.95	1.95	3.46	2.53
Yangzhou	1464	0.94	2.74	4.30	3.15
Zhenjiang	1464	0.95	0.91	2.65	1.92
Taizhou	1464	0.94	1.25	2.96	2.10
Suqian	1464	0.93	1.78	3.45	2.65
